# Trees and shrubs of the tropical dry forest of the Magdalena river upper watershed (Colombia)

**DOI:** 10.3897/BDJ.7.e36191

**Published:** 2019-08-30

**Authors:** Luz Piedad Romero-Duque, Jesion H Rosero-Toro, Mateo Fernández-Lucero, Andrea Simbaqueba-Gutierrez, Caterinne Pérez

**Affiliations:** 1 Universidad de Ciencias Aplicadas y Ambientales, Bogotá, Colombia Universidad de Ciencias Aplicadas y Ambientales Bogotá Colombia; 2 Universidad Surcolombiana, Neiva, Colombia Universidad Surcolombiana Neiva Colombia; 3 Universidad de los Andes, Bogotá, Colombia Universidad de los Andes Bogotá Colombia; 4 Universidad Pedagógica y Tecnológica de Colombia, Tunja, Colombia Universidad Pedagógica y Tecnológica de Colombia Tunja Colombia; 5 Independent Consultor, Bogotá, Colombia Independent Consultor Bogotá Colombia

**Keywords:** Plant diversity, secondary forests, tropical dry forest.

## Abstract

**Background:**

We describe the database of trees and shrubs of tropical dry forest patches of the Magdalena upper river basin in Colombia, preserved in the Herbarium of Universidad de Ciencias Aplicadas y Ambientales. The dataset includes 211 taxa, from which 156 were identified to species. We reported 48 families and 137 genera. The most species rich and abundant families were Fabaceae and Rubiaceae and the most abundant species was *Talisia stricta* (Sapindaceae). We found differences in diversity between north and south zones of the study area.

**New information:**

The Magdalena river upper watershed region is an important tropical dry forest conservation area. Twenty nine species and 4 genera recorded in this study have not been reported in previous reviews of the region. Additionally, *Oxandra espintana* is reported in literature as critically endangered and *Aspidosperma polyneuron* is reported as endangered, but there are no studies about their conservation status in the region. Our results suggest the strong need to develop additional inventories of plants that contribute to the knowledge of the plant diversity of this ecosystem in the region and studies of their conservation status.

## Introduction

Tropical dry forests (TDF) correspond to a complex and fragile ecosystem. This complexity is due to evaporation exceeding precipitation and there being one or two periods of drought which may last between 4 and 6 months/year, resulting in the defoliation of part of the vegetation by water stress (Janzen 1988, Murphy and Lugo 1986). Therefore, TDF has a great diversity of life forms, mixing deciduous and evergreen species with complex ecophysiological patterns (Dirzo et al. 2011a, Pennington et al. 2009). TDF has higher species richness in Mexico, Bolivia, Paraguay and Argentina (Gentry 1995, Olson and Dinerstein 1988), countries located in sites which challenge the pattern of increasing diversity when approaching the Ecuadorian line (Chazdon and Denslow 2002). Additionally, TDF has greater species richness thedrier they are, as in Mexico and Bolivia (Gentry 1995). Moreover, TDF has a high number of endemic species (Linares-Palomino et al. 2011) and provides a wide range of ecosystem services to human beings (Balvanera et al. 2012, Maass et al. 2005).

TDF is one of the most threatened ecosystems by human activity around the world (Hoekstra et al. 2004, Miles et al. 2006). There have been reports of strong erosional processes and loss of natural cover of TDF (Portillo-Quintero and Smith 2018), including its disappearance from some regions of Central and South America (Mares et al. 1985, Janzen 1988). This is because the TDF distribution area coincides with regions suitable for livestock and agriculture (Hoekstra et al. 2004) and where firewood and wood removal activities are practised. Portillo-Quintero and Sánchez-Azofeifa (2010) reported that 72% of TDF has been lost from North and Central America, whereas in South America, 60% has been lost.

Recently, research has paid more attention to TDF. However, studies on its structure and diversity are not evenly distributed in the Neotropics. Most of the studies have been concentrated in a few countries such as Mexico (e.g. López-Martínez et al. 2013, Dzib-Castillo et al. 2014, Palacios-Wassenaar et al. 2018, Silva Aparicio et al. 2018, Silva-Aparicio et al. 2018) and Brazil (e.g. Barbosa et al. 2012, Apgaua et al. 2014, Silva et al. 2014, Lima and Coelho 2015, de Queiroz et al. 2017, Rocha et al. 2017). For larger scales, Dirzo et al. (2011b) developed a regional synthesis for Latin America addressing aspects of the TDF ecology.

Colombia is one of those countries where TDF is the most threatened and least studied. Only 8% of TDF original distribution in the country remains (García et al. 2014). In their review of diversity and conservation status of TDF in Colombia, Pizano and García (2014) state that the available literature in Colombia consists of studies on local scale, concentrated mostly on the Caribbean coast (north of the country) and the Chicamocha (north-west), Cauca and Patia watersheds (south-west). Moreover, the authors state the largest number of samplings has taken place in the Caribbean and in the valley of Cauca river. The Magdalena river upper watershed has been less sampled and most of the samplings have been concentrated in the north area (e.g. Mendoza-C 1999, Figueroa-C and Galeano 2007, Frenández-Méndez et al. 2013, Villanueva et al. 2015, Melo-Cruz et al. 2016, Melo et al. 2017). Additionally, in the Magdalena river upper watershed, only 13% of its potential distribution remains (Romero-Duque et al., data not published). Our goal was to contribute to the knowledge of plant diversity of TDF of the Magdalena river upper watershed. This paper provides a large dataset for occurrences of trees and shrubs.

## Project description

### Title

Diversity and ecosystem services of Tropical dry forest of the upper Magdalena river basin, Colombia.

### Personnel

Romero-Duque, Luz Piedad. Universidad de Ciencias Aplicadas y Ambientales. Bogotá. Colombia.

Balvanera Levy, Patricia. Instituto de Investigación en Ecosistemas y Sostenibilidad. UNAM. Morelia. México.

Jaramillo Luque, Víctor J. Instituto de Investigación en Ecosistemas y Sostenibilidad. UNAM. Morelia. México.

### Study area description

**Figure 1.**
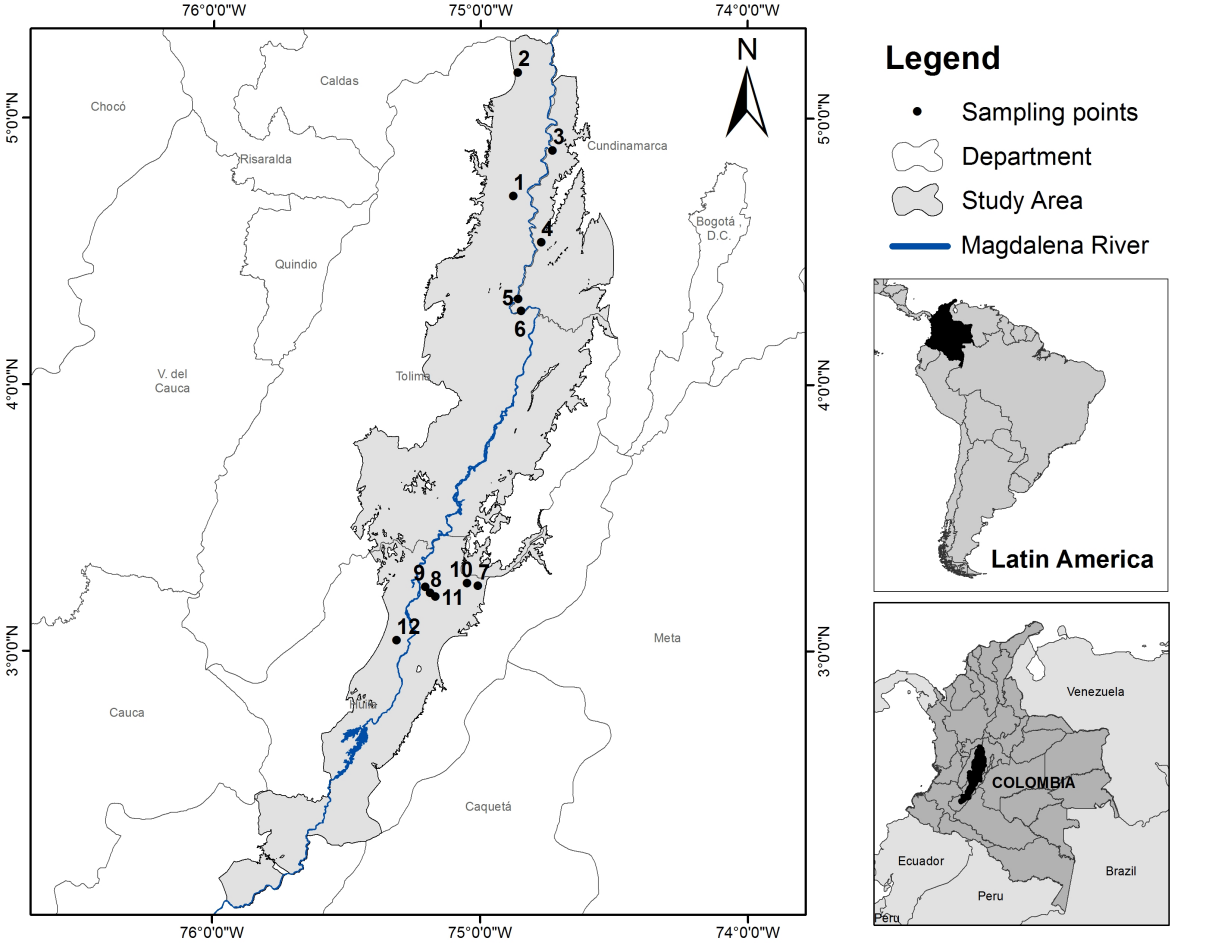
Distribution of tropical dry forest patches in the Magadalena river upper watershed (Colombia).

To determine the study area, we follow the Sánchez-Azofeifa et al. (2005) definition of TDF: "*this ecosystem is located in areas with an average temperature of 25ºC, annual precipitation between 700 and 2000 mm and with 3 o more dry months/year (less than 100 mm/month)*". Additionally, we considered the suggestion of Repizzo and Devia (2008) which states that, in Colombia, TDF is located below 1000 m asl. The study area is located in the south-western zone of the country, between the Central and Eastern mountain chain, on the geographical axis of the valley of the Magdalena river (Fig. 1). This area has an average annual rainfall of 1307 mm/year, with a bimodal precipitation regime (March-April; September-December) and an average temperature of 27°C. The study area has two zones of precipitation clearly defined. The north zone where precipitation varies from 1161 to 1431 mm/year and the south zone where most of the area varies from 730 to 1314 mm/year (data were obtained from the 30-year time series proportioned by IDEAM) (Table 1)

### Funding

Ecopetrol and Universidad de Ciencias Aplicadas y Ambientales.

## Sampling methods

### Sampling description

We selected twelve TDF patches according to their size, accessibility and owner's permission. Half of the patches were in Tolima and Cundinamarca departments (north zone of the study area) and the other half was in Huila department (south zone of the study area). In each site, we established ten 50 m x 2 m transects (0.1 ha), at least 7.5 m apart from each other at each site.

### Quality control

All the materials were processed following the standardised procedures for herbaria described by Forman and Bridson (1989). Taxonomic identification was made by botanical experts with the help of clues, texts (Gentry 1993, Mabberley 1997, Vargas 2002, Vargas 2012, Pizano and García 2014) and Flora Neotropica), papers (Villanueva et al. 2015, Ballesteros-Correa et al. 2019), web pages (Bernal et al. 2019, UDBC and Tropics) and documents with original botanical descriptions. Scientific names and all taxonomic validation were handled according to the standards of The Plant List (http://www.theplantlist.org) and APGIV (http://www.mobot.org/MOBOT/research/APweb/).

### Step description

According to the criteria of Font Quer (1979), two life forms were recognised: shrub (woody individual less than 5 m tall that branches from the base at 1.5 to 4.9 m) and tree (woody individual that had a shaft of ≥ 5 m in height), all the individuals rooted within the transect and having ≥ 1 cm of DAP, were measured (DBH, height) and were identified as fully as possible to species. We registered data as common name, form of growth, vegetative and reproductive characteristics of aroma, colour, exudate, indument and glands and made their respective photographic records. We packed the material in plastic bags for easy handling and then we put them in botanical presses. We collected flowers (when possible) and stored them in bottles with glycerine. We entered field data with the Darwin Core format and, with the advisory team of SiB Colombia, the database was published.

## Geographic coverage

### Description

Enpoints: 74°50'35"W; 5°18’40"N - 74°34'5"W, 3°53'10"N and 74°43'48"W, 3°17'31"N - 75°56'13"W, 2°2'60"N.

## Taxonomic coverage

### Description

The dataset contains a total of 655 tagged individuals. We found 211 taxa (48 families and 137 genera), from which 156 were identified to species (see data resource). Some individuals of 44 genera remained unidentified. This is mainly related to the lack of appropriate material (e.g. flowers) to provide a definite determination. Three species of Cactaceae family were included in the database.

For the total study area, Fabaceae and Rubiaceae were the most species rich and the most abundant (individuals sampled) families (Table 2). *Talisia stricta* (Sapindaceae) was the most abundant species recorded (35 individuals). The north zone was the most diverse (species number) (Table 2). We found 177 species, 120 genera and 43 families, whereas, in the south zone, we found 65 species, 47 genera and 26 families (Table 3). These differences could be due to the precipitation being higher in the north zone than in the south zone. Moreover, the north zone is a transition zone between tropical dry forest and tropical humid forest, which would explain the greater diversity, as well as the presence of some species representative of wet and moist forests in tropical dry forests (e.g. species from Bactris, Herrania, Monilicarpa, Posoqueria, Preslianthus, Swartzia and Trichilla genera). These species have been previous reported in tropical dry forest in Colombia (e.g. Pizano and García 2014, Villanueva et al. 2015, Ballesteros-Correa et al. 2019, Bernal et al. 2019).

**Figure 2.**
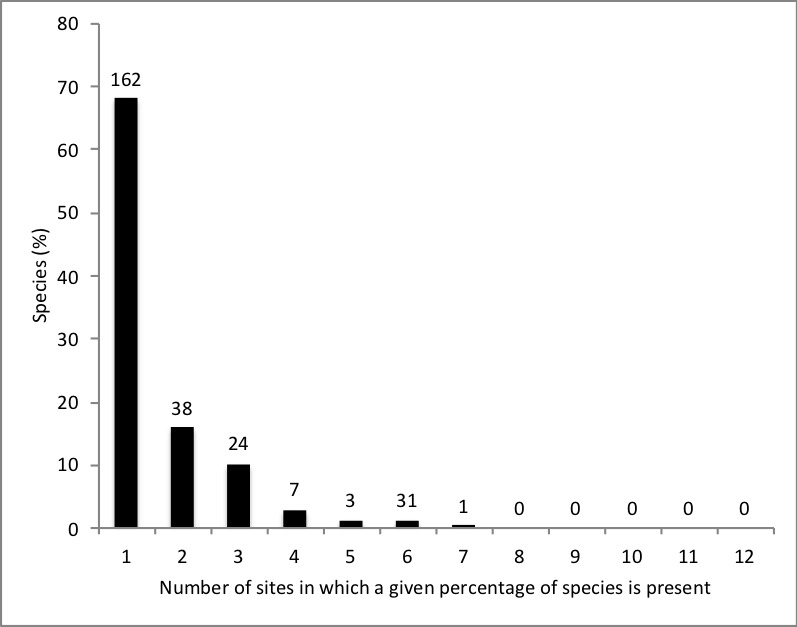
Frequency distribution of species according to the number of sites in which they occur. Number of species found within each category is shown above each bar.

The frequency distribution of the number of species amongst study sites was very skewed (Fig. 2). Sixty four percent of the species appeared in a single site, whereas 18% of the species appeared in two of the 12 sites. No species appeared in more than 5 sites. *Astronium graveolens* Jacq., *Casearia corymbosa* Kunth and *Randia armata* DC. were present in five sites (Table 4, Fig. 2). Only one species of the sampled species with a higher importance value index (relative abundance, relative density, relative frequency) was shared between north and south zones of the study area (Table 5). Our results coincide with those of Apgaua et al. (2014), Balvanera et al. (2002), Banda-R et al. (2016), amongst others, who reported a high variation in the richness and composition of species amongst TDF sites. Our results confirm the suggestion of Banda-R et al. (2016), about the need for multiple protected areas of TDF in the inter-Andean valleys.

## Temporal coverage

### Notes

Sep 2014 – End 2015

## Collection data

### Collection name

Colección biológica U.D.C.A

### Collection identifier

Registro Nacional de Colecciones Biológicas 51

### Parent collection identifier

UDCA

### Specimen preservation method

Drying and Pressing

## Usage rights

### Use license

Creative Commons Public Domain Waiver (CC-Zero)

## Data resources

### Data package title

Lista de árboles y arbustos del Bosque tropical seco del valle alto del río Magdalena, Colombia

### Resource link

https://www.gbif.org/dataset/39a96bb1-2fbd-4994-92f3-c1fc79b1bba3

### Alternative identifiers

https://doi.org/10.15472/hf3wnp

### Number of data sets

1

### Data set 1.

#### Data set name

Lista de árboles y arbustos del Bosque tropical seco del valle alto del río Magdalena, Colombia

#### Data format

Darwin Core Archive DwC-A

#### Number of columns

53

#### 

**Data set 1.** 

Column label	Column description
basisOfRecord	Specific nature of data record
catalogNumber	Identifier for the record within dataset
class	Scientific name of the class in which the taxon is classified
collectionCode	Name identifying the dataset from which the record was derived
collectionID	Identifier for the dataset from which the record was derived
continent	Name of the continent in which location occurs
country	Name of the country in which location occurs
countryCode	Standard code for the country in which location occurs
county	Name of the next smaller administrative region than country in which location occurs
dateIdentified	Date on which the subject was identified
decimalLatitude	Geographic latitude where occurrence was recorded
decimalLongitude	Geographic longitude where occurrence was recorded
eventDate	Date-time when the occurrence was recorded
family	Scientific name of the family in which the taxon is classified
genus	Scientific name of the genus in which the taxon is classified
geodeticDatum	Ellipsoid, geodetic datum or SRS, upon which the geographic coordinates are based
georeferencedBy	List of people's names who determined the georeference for the location
habitat	Habitat type where occurrence was registered
identificationQualifier	Brief phrase to express the determiner's doubts about the identification
identifiedBy	List of people's names who assigned the taxon to the subject
institutionCode	Name in use by the institution having custody of the object(s) referred to in the record
institutionID	Identifier for the institution having custody of the object(s) referred to in the record
kingdom	Scientific name of the kingdom in which the taxon is classified
language	Language of the resource
licence	Legal document giving official permission to do something with the resource
locality	Specific description of the place
locationID	Identifier for the set of location information
maximumElevationInMetres	Upper limit of the range of elevation
minimumElevationInMetres	Lower limit of the range of elevation
municipality	Name of the next smaller administrative region than county in which the location occurs
occurrenceID	Identifier for the occurrence
order	Scientific name of the order in which the taxon is classified
phylum	Scientific name of the phylum in which the taxon is classified
previousIdentifications	List of previous assignments of names to the organism
recordedBy	List of people's names responsible for recording the original occurrence
sampleSizeUnit	Unit of measurement of the sample size
sampleSizeValue	Measurement of sample size
samplingEffort	Amount of effort expended
samplingProtocol	Description of the method used
scientificName	Name of lowest level taxonomic rank that was determined
scientificNameAuthorship	Authorship information for the scientificName
specificEpithet	Name of the species epithet
stateProvince	Name of the next smaller administrative region than country in which the location occurs
taxonomicStatus	Status of the use of the scientificName as a label for a taxon linked to http://www.tropicos.org
taxonRank	Taxonomic rank of the most specific name in the scientificName
type	Kind of description
verbatimCoordinates	The verbatim original spatial coordinates of the Location
verbatimCoordinateSystem	The spatial coordinate system for the verbatimLatitude and verbatimLongitude or the verbatimCoordinates of the Location
verbatimElevation	The original description of the elevation of the Location
verbatimLocality	The original textual description of the place
verbatimSRS	The ellipsoid, geodetic datum or SRS upon which coordinates given in verbatimCoordinates are based
verbatimTaxonRank	Taxonomic rank of the most specific name in the scientificName as it appears in the original record
vernacularName	A common or vernacular name

## Figures and Tables

**Table 1.** Description of the sampled sites in the Magdalena river upper watershed (Colombia).

Zone	Department	Site	Latitude	Longitude	Elevation (m asl)	Total rainfal (mm)	Mean temperature (ºC)
North	Tolima	1	4.709891	-74.876878	300	1388	27
North	Tolima	2	5.174831	-74.861015	463	1811	25
North	Cundinamarca	3	4.882335	-74.730283	264	1431	28
North	Cundinamarca	4	4.536165	-74.772804	395	1161	27
North	Cundinamarca	5	4.322327	-74.860534	289	1345	27
North	Cundinamarca	6	4.282726	-74.850271	301	1345	27
Sur	Huila	7	3.245542	-75.010028	713	730	26
Sur	Huila	8	3.219611	-75.187444	417	1314	27
Sur	Huila	9	3.244972	-75.1945	374	1314	27
Sur	Huila	10	3.256278	-75.056278	549	1314	28
Sur	Huila	11	3.204629	-75.166832	413	1314	27
Sur	Huila	12	3.040488	-75.312614	507	1216	28

**Table 2.** a. Five families with the most species richness and number of individuals and b. Five most abundant species in tropical dry forest of the Magdalena river upper watershed (Colombia).

**Family**	**No. species**	**Family**	**No. Individuals**	**Species**	**No. Individuals**
Fabaceae	30	Fabaceae	108	Talisia stricta Triana & Planch. ex Radlk.	35
Rubiaceae	19	Rubiaceae	58	Casearia corymbosa Kunth	22
Euphorbiaceae	12	Sapindaceae	47	Astronium graveolens Jacq. - Cordia alliodora (Ruiz & Pav.) Oken	20
Bignoniaceae	10	Salicaceae	39	Guatteria Ruiz & Pav.	15
Annonaceae	8	Apocynaceae	27	Swartzia trianae Benth.	1

**Table 3.** Total number of families, genera and species of tropical dry forest in north and south zones of Magdalena river upper watershed (Colombia).

	**Family**	**Genera**	**Species**	**Individuals**
North zone	43	120	177	533
South zone	26	47	65	122
Total	48	137	211	655

**Table 4.** Species distribution amongst the twelve tropical dry forest patches in the Magdalena river upper watershed (Colombia).

**Species**	**Study site**											
	**1**	**2**	**3**	**4**	**5**	**6**	**7**	**8**	**9**	**10**	**11**	**12**
*Acacia farnesiana* (L.) Willd.											X	
*Acacia polyphylla* DC.	X											
*Acacia tortuosa* (L.) Willd.								X				
*Acalypha diversifolia* Jacq.		X										
*Acanthocereus tetragonus* (L.) Hummelinck			X									
*Achatocarpus nigricans* Triana				X		X					X	
*Adenocalymma patulum* (Miers) L.G.Lohmann			X									
*Albizia guachapele* (Kunth) Dugand		X										
*Ampelocera* Klotzsch							X					
*Amyris pinnata* Kunth					X	X						
*Amyris sylvatica* Jacq.				X			X					
Anacardiaceae				X								
*Anacardium excelsum* (Kunth) Skeels		X										
*Annona edulis* (Triana & Planch.) H.Rainer		X										
*Annona* L.				X								
*Apeiba glabra* Aubl.		X										
*Aphelandra glabrata* Willd. ex Nees				X		X						
Apocynaceae				X								
*Ardisia foetida* Willd. ex Roem & Schult.		X										
*Ardisia* Sw.						X						
*Aspidosperma cuspa* S.F.Blake ex Pittier	X						X			X		
*Aspidosperma desmanthum* Benth. ex Müll.Arg.		X										
*Aspidosperma polyneuron* Müll.Arg.				X	X	X						
*Astronium graveolens* Jacq.	X		X	X	X	X				X		
*Astronium* Jacq.		X										
*Bactris major* Jacq.		X										
*Bactris pilosa* Karst.		X										
*Banisteriopsis* C.B.Rob. ex Small									X			
*Bauhinia guianensis* Aubl.										X		
*Bauhinia* L.							X					
*Beilschmiedia sulcata* (Ruiz & Pav.) Kosterm.				X								
Bignoniaceae				X								
*Brownea ariza* Benth.		X		X		X						
*Bunchosia pseudonitida* Cuatrec.		X	X	X	X							
*Bunchosia* Rich. ex Juss.												X
*Bursera* Jacq. ex L.										X		
*Bursera simaruba* Sarg.			X		X		X					
*Bursera tomentosa* Triana & Planch.					X							X
*Byttneria aculeata* Jacq.										X		
*Calliandra magdalenae* Benth.		X										
*Calliandra riparia* Pittier							X					
*Capparidastrum frondosum* (Jacq.) Cornejo & Iltis	X			X	X							
*Capparis* L.				X								
*Casearia corymbosa* Kunth	X			X	X	X			X			N
*Casearia* Jacq.	X	X				X						
*Casearia sylvestris* Sw.	X	X										
*Casearia tremula* (Griseb.) Griseb. ex C.Wright										X		
*Celtis trinervia* Lam.				X	X							
*Cereus hexagonus* Mill.			X									
*Cestrum* L.				X								
*Cestrum mutisii* Willd. ex Roem. & Schult.					X							
*Chiococca alba* Hitchc.						X						
*Chloroleucon mangense* Britton & Rose	X		X									
*Chomelia spinosa* Jacq.		X										
*Chrysochlamys* Poepp. & Endl.		X										
*Cinchona* L.				X	X	X						
*Cinnamomum triplinerve* (Ruiz & Pav.) Kosterm.		X										
*Citharexylum sulcatum* Moldenke					X	X						
*Clusia schomburgkiana* Benth. ex Engl.							X					
*Coccoloba obovata* Kunth		X										
*Colubrina* Rich. ex Brongn.				X								
*Cordia alliodora* (Ruiz & Pav.) Oken	X			X								
*Cordia bifurcata* Roem. & Schult.				X								
*Cordia dentata* J.L.M.Poiret											X	
*Cordia* L.												X
*Cordia macrocephala* (Desv.) Kunth										X		
*Coutarea hexandra* (Jacq.) K.Schum.								X				
*Croton argyrophyllus* Kunth						X						
*Croton caracasanus* Pittier							X					
*Croton ferrugineus* Kunth				X	X							
*Croton fragrans* Kunth		X	X									
*Croton glabellus* L.						X	X					
*Croton* L.		X			X		X					
*Croton leptostachyus* Kunth					X							
*Croton schiedeanus* Schltdl.										X		
*Cupania* L.		X										
*Cupania latifolia* Kunth				X	X							
*Cupania papillosa* Radlk.				X								
*Cynophalla flexuosa* J.Presl	X		X				X					
*Erythrina* L.				X								
*Erythroxylum cassinoides* Planch. & Linden		X										
*Erythroxylum hondense* Kunth				X								
*Erythroxylum macrophyllum* Cav.				X	X	X						
*Erythroxylum ulei* O.E.Schulz		X										
*Esenbeckia* Kunth							X					
*Eugenia* Mich. ex L.	X			X		X						
*Eugenia procera* Poir.		X					x					
Fabaceae				X								
*Faramea capillipes* Müll.Arg.		X										
*Forsteronia* G.Mey.								X				
*Garcinia madruno* (Kunth) Hammel		X										
*Genipa americana* L.		X										
*Guapira costaricana* (Standl.) Woodson			X				X					X
*Guapira pubescens* (Kunth) Lundell										X		
*Guatteria hirsuta* Ruiz & Pav		X										
*Guatteria* Ruiz & Pav.				X	X	X						
*Guazuma ulmifolia* Lam.	X			X	X	X			X			
*Guettarda malacophylla* Standl.							X			X		X
*Gustavia santanderiensis* R.Knuth		X		X		X						
*Gustavia verticillata* Miers				X		X						
*Handroanthus chrysanthus* (Jacq.) S.O.Grose	X											
*Helianthostylis sprucei* Baill.		X										
*Helicostylis* Trécul				X								
*Helicteres baruensis* Jacq.	X						X			X		
*Herrania laciniifolia* Goudot		X										
*Hiraea* Jacq.	X						X					
*Hirtella americana* L.		X										
*Inga densiflora* Benth.		X										
*Jacaranda caucana* Pittier		X										
*Jatropha gossypiifolia* L.										X		
*Laetia americana* L.	X											
*Lunania parviflora* Spruce ex Benth.					X							
*Machaerium capote* Triana ex Dugand	X	X		X	X							
*Machaerium goudotii* Benth.				X	X							
*Machaerium microphyllum* Standl.	X											
*Machaerium* Pers.			X	X	X							
*Machaonia acuminata* Humb. & Bonpl.										X		
*Maclura tinctoria* (L.) D.Don ex Steud.	X							X				
*Magnoliophyt*a				X	X	X						
*Malpighia glabra* L.				X		X						
*Malpighia* L.										X		
Malpighiaceae				X			X					
Malvaceae					X		X					
*Manihot carthagenensis* (Jacq.) Müll.Arg.							X					
*Maripa* Aubl.								X				
*Marsdenia xerohylica* Dugand				X								
*Matayba* Aubl.		X					X					
*Memora patula* Miers					X	X						
*Miconia spicellata* Bonpl. ex Naudin		X										
*Monilicarpa tenuisiliqua* (Jacq.) Cornejo & Iltis				X								
*Morisonia americana* L.				X								
*Mouriri colombiana* Morley		X										
*Mussatia* Bureau ex Baill.								X				
*Myrcia* DC.		X		X		X						X
*Neea divaricata* Poepp. & Endl.				X	X							
*Neea* Ruiz & Pav.				X								
*Ocotea veraguensis* (Meisn.) Mez		X										
*Onoseris purpure*a				X								
*Ouratea* Aubl.	X											
*Oxandra espintana* (Spruce ex Benth.) Baill.	X											
*Oxandra venezuelana* R.E.Fr.		X										
*Paullinia densiflora* Sm.									X			
*Paullinia nitida* Kunth				X								
*Petrea rugosa* Kunth	X	X										
*Picramnia sphaerocarpa* Planch.		X										
*Piper marginatum* Jacq.		X										
*Piptocoma discolor* (Kunth) Pruski		X										
*Pisonia aculeata* L.	X											
*Pithecellobium dulce* Benth.			X	X	X						X	
*Pithecellobium lanceolatum* Benth.					X							
*Platymiscium hebestachyum* Benth.	X		X	X	X							
*Platymiscium pinnatum* (Jacq.) Dugand				X	X							
*Pleonotoma variabilis* Miers				X								
*Posoqueria latifolia* Roem. & Schult.		X										
*Pouteria* Aubl. & Eyma	X			X		X						
*Pradosia colombiana* (Standl.) T.D.Penn. ex T.J.Ayers & Boufford		X										
*Preslianthus detonsus* (Triana & Planch.) Iltis & Cornejo				X	X	X						
*Prosopis juliflora* DC.			X		X							
*Protium* Burm.f.						X						
*Protium sagotianum* Marchand		X										
*Pseudobombax septenatum* (Jacq.) Dugand		X				X		X				
*Pseudolmedia laevis* (Ruiz & Pav.) J.F.Macbr.				X								
*Psidium guineense* Sw.				X	X							
*Psychotria carthagenensis* Jacq.		X										
*Psychotria micrantha* Kunth		X										
*Quadrella odoratissima* (Jacq.) Hutch.			X		X							
*Raimondia* Saff.					X							
*Randia aculeata* L.			X	X	X					X	X	
*Randia armata* DC.		X	X	X	X				X			
*Randia calycina* Cham.				X	X	X						
*Randia* L.				X	X							
*Rollinia* A.St.-Hil.				X								
*Rondeletia pubescens* Kunth		X		X								X
Rubiaceae				X								
*Ruprechtia ramiflora* C.A.Mey.					X							
*Sapium glandulosum* (L.) Morong		X										
*Schaefferia frutescens* Jacq.										X		
*Schnella* Raddi	X											
*Securidaca* L.												X
*Senegalia* Raf.	X	X					X					
*Senegalia riparia* (Kunth) Britton & Rose								X				
*Sideroxylon celastrinum* (Kunth) T.D.Penn.										X		
*Simira cordifolia* (Hook.f.) Steyerm.							X					
*Simira rubescens* (Benth.) Bremek. ex Steyerm.		X										
*Solanum arboreum* Humb. & Bonpl. ex Dunal				X		X						
*Solanum* L.				X					X			
*Sorocea* A.St.-Hil.				X								
*Spondias radlkoferi* Donn.Sm.		X										
*Stemmadenia grandiflora* (Jacq.) Miers				X								
*Stenocereus griseus* (Haw.) Buxb.			X									
*Swartzia* Schreb.		X		X		X						
*Swartzia simplex* Spreng.		X										
*Swartzia trianae* Benth.	X		X									
*Tabebuia chrysantha* (Jacq.) Nicholson					X							
*Tabebuia* Gomes ex DC.		X										
*Tabebuia ochracea* (Cham.) Standley	X		X	X								
*Talisia stricta* Triana & Planch. ex Radlk.	X			X		X						
*Trichilia acuminata* C.DC.				X		X						
*Trichilia elegans* A.Juss.	X											
*Trichilia oligofoliolata* M.E.Morales-Puentes	X		X									
*Trichilia pallida* Sw.		X		X	X	X						
*Triplaris americana* L.	X			X	X	X						
*Triumfetta acuminata* Kunth				X								
*Trophis* P.Browne		X										
*Valeriana* L.				X								
*Zanthoxylum fagara* Sargent				X	X			X		X		
*Zanthoxylum* L.					X	X		X	X			X
*Zanthoxylum quinduense* Tul.				X								
*Zanthoxylum rigidum* Humb. & Bonpl. ex Willd.	X			X	X							X
*Zygia inaequalis* Pittier		X										
Total general	34	64	21	78	47	36	23	10	7	18	5	11

**Table 5.** The ten species with the highest importance value index in north and south zones of the tropical dry forest of Magdalena river upper watershed (Colombia).

**Species**	**North**	**Species**	**South**
Randia armata DC.	0.74	Guettarda malacophylla Standl.	1.01
Machaerium capote Triana ex Dugand	0.73	Aspidosperma cuspa S.F.Blake ex Pittier	0.74
Bunchosia pseudonitida Cuatrec.	0.72	Casearia corymbosa Kunth	0.74
Astronium graveolens Jacq.	0.66	Helicteres baruensis Jacq	0.72
Casearia corymbosa Kunth	0.63	Zanthoxylum L.	0.72
Talisia stricta Triana & Planch. ex Radlk.	0.57	Guapira costaricana (Standl.) Woodson	0.69
Platymiscium hebestachyum Benth.	0.57	Banisteriopsis C.B.Rob. ex Small	0.38
Pouteria Aubl. & Eyma	0.56	Coutarea hexandra (Jacq.) K.Schum.	038
Casearia Jacq.	0.56	Forsteronia G.Mey.	0.38
Myrcia DC.	0.56	Malpighia L.	0.38
